# Identification of human pathogens isolated from blood using microarray hybridisation and signal pattern recognition

**DOI:** 10.1186/1471-2180-7-78

**Published:** 2007-08-14

**Authors:** Herbert Wiesinger-Mayr, Klemens Vierlinger, Rudolf Pichler, Albert Kriegner, Alexander M Hirschl, Elisabeth Presterl, Levente Bodrossy, Christa Noehammer

**Affiliations:** 1 Molecular Diagnostics, Austrian Research Centers GmbH – ARC, Mendelstrasse 1, A-2444 Seibersdorf, Austria; 2 Institute of Hygiene, Medical University of Vienna, Währinger Gürtel 18-20, A-1090 Vienna, Austria; 3 Division Infectious Diseases, Department of Medicine I, Medical University of Vienna, Währinger Gürtel 18-20, A-1090 Vienna, Austria; 4 Biogenetics and Natural Resources, Austrian Research Centers GmbH – ARC, Mendelstrasse 1, A-2444 Seibersdorf, Austria

## Abstract

**Background:**

Pathogen identification in clinical routine is based on the cultivation of microbes with subsequent morphological and physiological characterisation lasting at least 24 hours. However, early and accurate identification is a crucial requisite for fast and optimally targeted antimicrobial treatment. Molecular biology based techniques allow fast identification, however discrimination of very closely related species remains still difficult.

**Results:**

A molecular approach is presented for the rapid identification of pathogens combining PCR amplification with microarray detection. The DNA chip comprises oligonucleotide capture probes for 25 different pathogens including Gram positive cocci, the most frequently encountered genera of *Enterobacteriaceae*, non-fermenter and clinical relevant *Candida *species. The observed detection limits varied from 10 cells (e.g. *E. coli*) to 10^5 ^cells (*S. aureus*) per mL artificially spiked blood. Thus the current low sensitivity for some species still represents a barrier for clinical application. Successful discrimination of closely related species was achieved by a signal pattern recognition approach based on the k-nearest-neighbour method. A prototype software providing this statistical evaluation was developed, allowing correct identification in 100 % of the cases at the genus and in 96.7 % at the species level (n = 241).

**Conclusion:**

The newly developed molecular assay can be carried out within 6 hours in a research laboratory from pathogen isolation to species identification. From our results we conclude that DNA microarrays can be a useful tool for rapid identification of closely related pathogens particularly when the protocols are adapted to the special clinical scenarios.

## Background

Despite the continued progress in diagnosis and therapy of sepsis and septicemia mortality remains high. Current methods for the identification of microorganisms are based on the cultivation of the pathogens from blood with subsequent morphological and physiological characterisation [1]. More than 95 % of all bloodstream infections are caused by only 15 different genera of bacteria. *Staphylococci *and *E. coli *account for more than 50 % of the infections [2-6]. Treatment within six hours after the first symptoms of bacteremia is crucial otherwise the infection may progress to severe sepsis.

Automated blood culture systems such as BacT/Alert and BACTEC9240 are the standard cultivation techniques in modern clinical practice. False negative results occur periodically due to inappropriate growth conditions or antimicrobial treatment. Blood cultures without detectable microbial growth are manually subcultured, and subsequently positive results were obtained in 3 to 40 % of the cases depending on the detection method [5,7,8]. Conventional diagnostic methods last at least 24 hours due to their requirement for microbial growth rates. In general the detection and identification is a process taking two days for most organisms or even longer for fastidious organisms [9-11]. In contrast to this, DNA-based methods meet the needs for a fast, reliable and thereby life-saving diagnosis [12,13]. Methods based on PCR amplification and subsequent hybridisation of fluorescent probes seem to be most promising approaches for early diagnosis [1,14]. Several molecular methods, including the utilisation of fluorescently labelled probes, have been tried for the detection of clinical pathogens. Fluorescent *in situ *hybridisation (FISH), PCR, real time PCR, single strand conformation polymorphism (SSCP), and oligonucleotide microarrays have been adapted for the identification of isolated microorganisms from bacteraemia patients [3,15-23]. All DNA based pathogen identification methods for bloodstream infections comprise the detection of viable and dead cells as well as released microbial DNA of already lysed cells. This can lead to misinterpretation of molecular biology based detection methods on bacteria counts compared to pathogen numbers found in patients' blood using cultivation methods where e.g. only up to 10^2 ^pathogens per mL blood were reported in cases of severe sepsis. In contrast, applying quantitative RT-PCR, the density of gram positive or negative microorganisms in blood was found in a range of 10^4 ^to 10^7 ^per mL in bacteremia patients. Hackett even showed a concentration peak in severe cases of septicaemia to a maximum of 1.8 × 10^9 ^bacteria per mL [24-26]. Heininger *et al*. (1999) demonstrated the advantage of PCR detection of preceding antibiotic treatment in a rat model. Whereas the detection rate of classical blood cultures falls to 10 % within 25 min after intravenous administration of cefotaxime, the PCR detection rate is still 100% at that time [27].

The microarray technology has been described as a powerful tool to assess multiple parameters at the same time for various clinical scenarios such as pathogen identification of urinary tract infections (UTI), acute upper respiratory tract infections, periodontal pathogens and human intestinal bacteria. Microarrays are further applied for the analysis of microbial gene expression and diversity [28-32].

In this study a DNA microarray-based assay is presented which allows the identification of 25 different bloodstream infection relevant pathogens (bacteria and fungi) from whole blood samples. The array represents a further contribution towards a possible implementation of DNA chips in clinical diagnostics. The complete analysis can be done within 6 hours in a research laboratory starting with pathogen isolation from artificially-spiked blood to species identification. Such a rapid diagnostic test could support decisions for antibiotic treatments due to accurate discrimination of closely related species.

## Results

### Probe and array design

**Figure 1:**
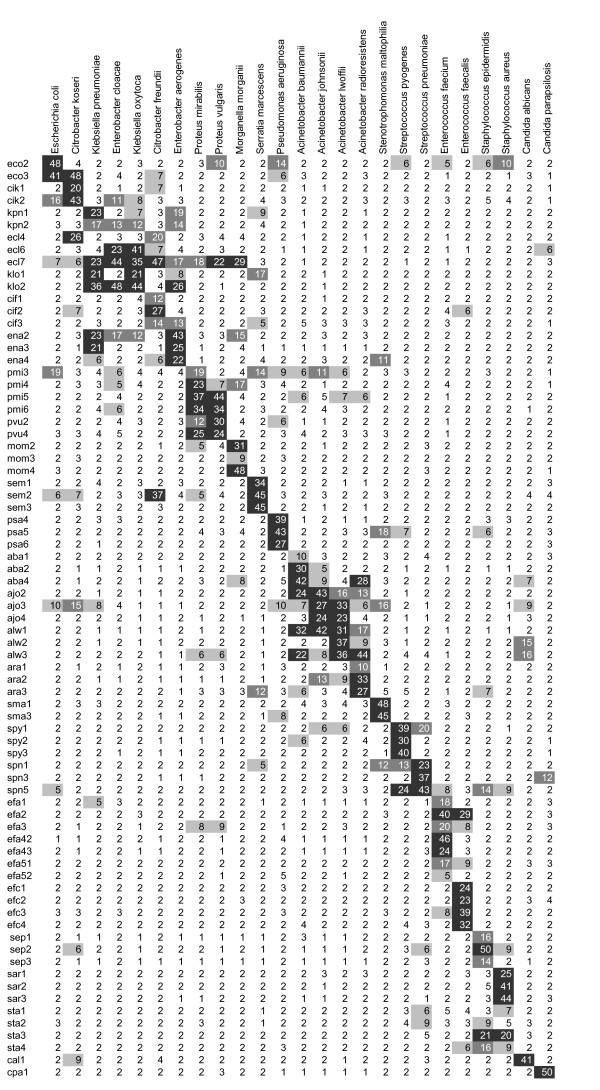
Normalised signal intensities of all hybridisation experiments listed by probe and species. The raw signal values were first normalised using quantile normalisation, and then averaged across spot-replicates and hybridisation-replicates (real values were divided by 1000 for better visualisation). No cut off value or signal limit was set in order to use absolute intensities for normalisation and table calculation. Background signals were subtracted prior to statistical evaluation. Background corrected hybridisation signals of 5001 – 10000, 10001 – 20000, and > 20001, are indicated in grey, dark grey and black, respectively. Normalised values lower than 5000 are not colour-coded. For calculations absolute values were used without defining a threshold that led to indication of low signals even when signals were flagged negative by the GenePix analysis software. Species are listed according to the phylogenetic relation of 16S and 18S rRNA sequences. Probes are sorted by species specificity. Abbreviations of probe names are listed in table 1.

Bacterial 16S rRNA sequences and 18S rRNA sequences of *Candida sp*. were imported into the arb software database and aligned to sequences present in the database. A phylogenetic tree of all species which can be identified with the newly developed microarray was calculated using the neighbour joining method. All in all 96 different DNA probes were designed using the arb software package. Additional probes were downloaded from the probeBase website [33]. More than 116 different probes were applied and separately evaluated in this study and 76 probes were further kept for the final set up (see table 1). The other ones were removed because of cross hybridisations or insufficiently specific signal responses. The hybridisation behaviour was found to be difficult to predict but calculating binding capacities by consideration of weighted mismatches still gives a very precise idea about overall species identification patterns (see table 2 and 3). However, different probes for identification of the same species still performed differently although they had equal *in silico *predicted behaviour, similar positions on the marker gene, similar G+C contents, lengths and melting temperatures (compare probes *ecl4*, *ecl6 *and *ecl7 *in table 1 and figure 1).

**Table 1:** List of probes used in this study

Specificity	Name	*E. coli Pos*.	Sequence [5' – 3']	Length bases	Tm°C	GC %	Ref.
*Ab. baumannii*	aba1	64	CAAGCTACCTTCCCCCGCT	19	60.3	63	this
	aba2	453	GTAACGTCCACTATCTCTAGGTATTAACTAAAGTAG	36	59.1	36	work
	aba4	1132	GCAGTATCCTTAAAGTTCCCATCCGAAAT	29	60.8	41	"
*Ab. johnsonii*	ajo2	620	TCCCAGTATCGAATGCAATTCCTAAGTT	28	60.1	39	"
	ajo3	979	GAAAGTTCTTACTATGTCAAGACCAGGTAAG	31	58.8	39	"
	ajo4	1114	CTTAACCCGCTGGCAAATAAGGAAAA	26	60	42	"
*Ab. lwoffii*	alw1	133	GAGATGTTGTCCCCCACTAATAGGC	25	60.4	52	"
	alw2	577	TGACTTAATTGGCCACCTACGCG	23	61	52	"
	alw3	637	CCCATACTCTAGCCAACCAGTATCG	25	59.9	52	"
*Ab*.	ara1	78	CGCTGAATCCAGTAGCAAGCTAC	23	59.1	52	"
*radioresistens*	ara2	450	GTCCACTATCCTAAAGTATTAATCTAGGTAGCCT	34	60.3	38	"
	ara3	1115	CCGAAGTGCTGGCAAATAAGGAAA	24	59.8	46	"
*Cb. freundii*	cif1	62	GCTCCTCTGCTACCGTTCG	19	58.2	63	"
	cif2	442	CCACAACGCCTTCCTCCTCG	20	61.1	65	"
	cif3	472	TCTGCGAGTAACGTCAATCGCTG	23	60.7	52	"
*Cb. koseri*	cik1	469	CGGGTAACGTCAATTGCTGTGG	22	59.9	55	"
	cik2	639	CGAGACTCAAGCCTGCCAGTAT	22	60	55	"
*Eb. cloacae*	ecl4	471	GCGGGTAACGTCAATTGCTGC	21	60.6	57	"
	ecl6	643	CTACAAGACTCCAGCCTGCCA	21	60	57	"
	ecl7	652	TACCCCCCTCTACAAGACTCCA	22	60	55	"
*Eb. aerogenes*	ena2	444	GGTTATTAACCTTAACGCCTTCCTCCT	27	60.2	44	"
	ena3	453	CAATCGCCAAGGTTATTAACCTTAACGC	28	60.4	43	"
	ena4	473	TCTGCGAGTAACGTCAATCGCC	22	60.8	55	"
*K. pneumoniae*	kpn1	61	GCTCTCTGTGCTACCGCTCG	20	60.7	65	"
	kpn2	203	GCATGAGGCCCGAAGGTC	18	58.9	67	"
*K. oxytoca*	klo1	81	TCGTCACCCGAGAGCAAGC	19	60.5	63	"
	klo2	633	CCAGCCTGCCAGTTTCGAATG	21	60	57	"
*E. coli*	eco2	448	GTAACGTCAATGAGCAAAGGTATTAACTTTACTCCCTTCC	40	61.9	40	31
	eco3	994	CCGAAGGCACATTCTCATCTCTGAAAACTTCCGTGGATG	39	65.6	49	31
*M. morganii*	mom2	121	GCCATCAGGCAGATCCCCATAC	22	60.9	59	this
	mom3	440	CTTGACACCTTCCTCCCGACT	21	59.7	57	work
	mom4	581	CATCTGACTCAATCAACCGCCTG	23	59.4	52	"
*P. mirabilis*	pmi3	247	GTCAGCCTTTACCCCACCTACTAG	24	59.8	54	"
	pmi4	444	GGGTATTAACCTTATCACCTTCCTCCC	27	60	48	"
	pmi5	625	CCAACCAGTTTCAGATGCAATTCCC	25	60.4	48	"
	pmi6	820	GTTCAAGACCACAACCTCTAAATCGAC	27	59.3	44	"
*P. vulgaris*	pvu2	179	CTGCTTTGGTCCGTAGACGTCA	22	60.3	55	"
	pvu4	1010	TTCCCGAAGGCACTCCTCTATCTCTA	26	61.9	50	"
*Pm. aerogenes*	psa4	585	GATTTCACATCCAACTTGCTGAACCA	26	59.9	42	"
	psa5	1136	TCTCCTTAGAGTGCCCACCCG	21	61.7	62	"
	psa6	1245	CGTGGTAACCGTCCCCCTTG	20	61	65	"
*Sr. marcescens*	sem1	62	CTCCCCTGTGCTACCGCTC	19	60.4	68	"
	sem2	439	CACCACCTTCCTCCTCGCTG	20	60.7	65	"
	sem3	460	GAGTAACGTCAATTGATGAGCGTATTAAGC	30	59.8	40	"
*Sm. maltophilia*	sma1	713	AGCTGCCTTCGCCATGGATGTTC	23	63.7	57	"
	sma3	1265	TGGGATTGGCTTACCGTCGC	20	61	60	"
*S. pneumoniae*	spn1	56	CTCCTCCTTCAGCGTTCTACTTGC	24	60.7	54	"
	spn3	201	GGTCCATCTGGTAGTGATGCAAGTG	25	60.9	52	"
	spn5	634	TCTTGCACTCAAGTTAAACAGTTTCCAAAG	30	60.1	37	"
*S. pyogenes*	spy1	175	ATTACTAACATGCGTTAGTCTCTCTTATGCG	31	60.2	39	"
	spy2	471	CTGGTTAGTTACCGTCACTTGGTGG	25	60.8	52	"
	spy3	623	TTCTCCAGTTTCCAAAGCGTACATTG	26	59.6	42	"
*Ec. faecium*	efa1	67	CAAGCTCCGGTGGAAAAAGAAGC	23	60.3	52	"
	efa2	208	CATCCATCAGCGACACCCGA	20	60.4	60	"
	efa3	1240	ACTTCGCAACTCGTTGTACTTCCC	24	60.8	50	"
	efa42	446	CCGTCAAGGGATGAACAGTTACTCTCATCCTTGTTCTTC	39	66.8	46	31
	efa43	1242	ATTAGCTTAGCCTCGCGACTTCGCAACTCGTTGTACTTC	39	69.3	49	31
	efa51	65	CTCCGGTGGAAAAAGAAGCGT	21	59	52	this
	efa52	82	CTCCCGGTGGAGCAAG	16	57	52	work
*Staphylococcus*	sta1	995	CTCTATCTCTAGAGCGGTCAAAGGAT	26	59	46	"
	sta2	1137	CAGTCAACCTAGAGTGCCCAACT	23	60	52	"
	sta3	1237	AGCTGCCCTTTGTATTGTCCATT	23	59	44	"
	sta4	1264	ATGGGATTTGCATGACCTCGCG	22	62	55	"
*Sta. aureus*	sar1	186	CCGTCTTTCACTTTTGAACCATGC	24	59	46	"
	sar2	230	AGCTAATGCAGCGCGGATC	19	59	58	"
	sar3	447	TGCACAGTTACTTACACATATGTTCTT	27	57	33	"
*Sta. epidermidis*	sep1	1005	AAGGGGAAAACTCTATCTCTAGAGGG	26	59	46	"
	sep2	983	GGGTCAGAGGATGTCAAGATTTGG	24	59	50	"
	sep3	993	ATCTCTAGAGGGGTCAGAGGATGT	24	60	50	"
*Ec. faecalis*	efc1	84	CCACTCCTCTTTCCAATTGAGTGCA	24	61	50	"
	efc2	176	GCCATGCGGCATAAACTGTTATGC	24	61	50	"
	efc3	193	CCCGAAAGCGCCTTTCACTCTT	22	62	55	"
	efc4	452	GGACGTTCAGTTACTAACGTCCTTG	25	59	48	"
*C. albicans*	cal1	-	CCAGCGAGTATAAGCCTTGGCC	22	61.2	59	62
*C. parapsilosis*	cpa1	-	TAGCCTTTTTGGCGAACCAGG	21	60.6	52	62

List of probes used in this study including their nucleotide sequences and some characteristics. Abbreviations: Ab: *Acinetobacter*, Cb: *Citrobacter*, Eb: *Enterobacter*, Ec: *Enterococcus*, E: *Escherichia*, K: *Klebsiella*, M: *Morganella*, P: *Proteus*, Pm: *Pseudomonas*, Sr: *Serratia*, Sm: *Stenotrophomonas*, S: *Streptococcus*, Sta: *Staphylococcus*, C: *Candida*

**Table 2:** Influence of mismatch position on weighted mismatch calculation

5' – 3' Position							
1	2	3	4	N	3	2	1
0.3	0.6	1.0	1.2	1.2	1.1	0.8	0.3

Weights for mismatches related to their position in the sequence. A single mismatch at the first position of the probe is weighted with 0.3 whereas mismatches at central positions of the probes were weighted highest with 1.2. These values were experimentally determined.

**Table 3:** Influence of mismatch type on weighted mismatch calculation

Probe	Target		Probe	Target		Probe	Target		Probe	Target	
A -	A	1.0	G -	A	1.0	C -	A	0.7	T -	C	1.0
-	C	0.4	-	G	1.0	-	C	1.0	-	G	1.0
-	G	1.2	-	T	1.0	-	T	1.0	-	T	1.0

Weights of mismatches due to the type of mismatched bases. A mismatch of adenine on the probe with cytosine on the target sequence is weighted with 0.4, whereas a mismatch of the same probe with a guanine in the target sequence is weighted with 1.2.

To determine optimal probe quantities on the microarray different probe concentrations (10, 20 and 50 μM) were printed in a defined buffer. Probe concentrations of 50 μM gave highest signals. Probe concentrations above 50 μM were not tested since they were already found in other microarray applications not to lead to further signal increase.

### Specificity

An *in silico *hybridisation matrix was generated with the Probe Match function in the ARB software package and the CalcOligo software. The modelled hybridisation behaviour of each probe was in good agreement with real experimental data (for comparison see fig. 1 and Additional file 1). Cross hybridisation within the *Enterobacteriaceae *family was already predicted by *in silico *calculations due to highly conserved 16S rRNA sequences and confirmed by experimental results. The calculated matrix showed similar hybridisation patterns as achieved through hybridisation assays (see Additional file 1). Due to the application of short oligonucleotide probes it was possible to discriminate sequences differing from each other by a single nucleotide. This was demonstrated using probe *ecl 6 *(starting at *E. coli *position 643, see table 1) that matches perfectly with the species *E. cloacae *and *K. oxytoca *generating average signal intensities of 23000, 41000 respectively. In contrast, hybridising amplified 16S rDNA of the species *E. aerogenes *and *K. pneumoniae *resulted in no detectable signal because of a nucleotide change at *E. coli *position 653 (cytosine is changed for a thymine in the bacterial genome). This mismatch was only weighted with 0.8 applying the parameters described in table 2 and 3.

Normalised signal values of 241 hybridisation experiments are summarised in Fig 1 and also supplied as table (see Additional file 2). The observed hybridisation values showed low coefficient of variation (CV) among the 6 replicate spots and between the different assays. The CV of all specific signals ranged from 2.4 % to 64.1 % for 80 % of the probes. High CV values resulted from experiments done at or just above the detection limit yielding only faint signals (e.g. when 10 *E. coli *or 10^2 ^*K. pneumoniae *cells were used per assay. For further details see the paragraph "Sensitivity"). Since in clinical routine the possibility of little initial bacterial quantities is rather high reliable result interpretation must be guaranteed even at low bacterial loads. This represents a limitation of short oligonucleotide microarrays since they are at risk to lead to a misinterpretation of results below or also near the detection limit. Species that were not used at the detection limit, such as *S. marcescens *or *S. maltophilia*, gave lower CV values ranging from 5 % to 18 %. As expected from CalcOligo analysis, cross-hybridisations of individual probes occurred within the *Enterobacteriaceae *family especially in the group of *Klebsiella-Enterobacter-Citrobacter*. However, specific signal patterns could be assigned to each species enabling the identification of cultures at species level. For *Acinetobacter *and *Proteus *reliable identification is only guaranteed at genus level which is sufficient for most clinical applications.

### Sensitivity

Limits of bacterial detection (LOD) were assessed with spiked blood samples and pure cultures using dilution series from 10^8 ^to 10^0 ^bacteria per mL from selected gram positive and gram negative bacterial species. The detection limit in pure cultures was lower than in spiked blood due to PCR interference of blood components. PCRs carried out from pure cultures were found to amplify DNA down to 10^3 ^cells per assay resulting in a clearly visible band on a 1.5% agarose gel.

**Figure 2:**
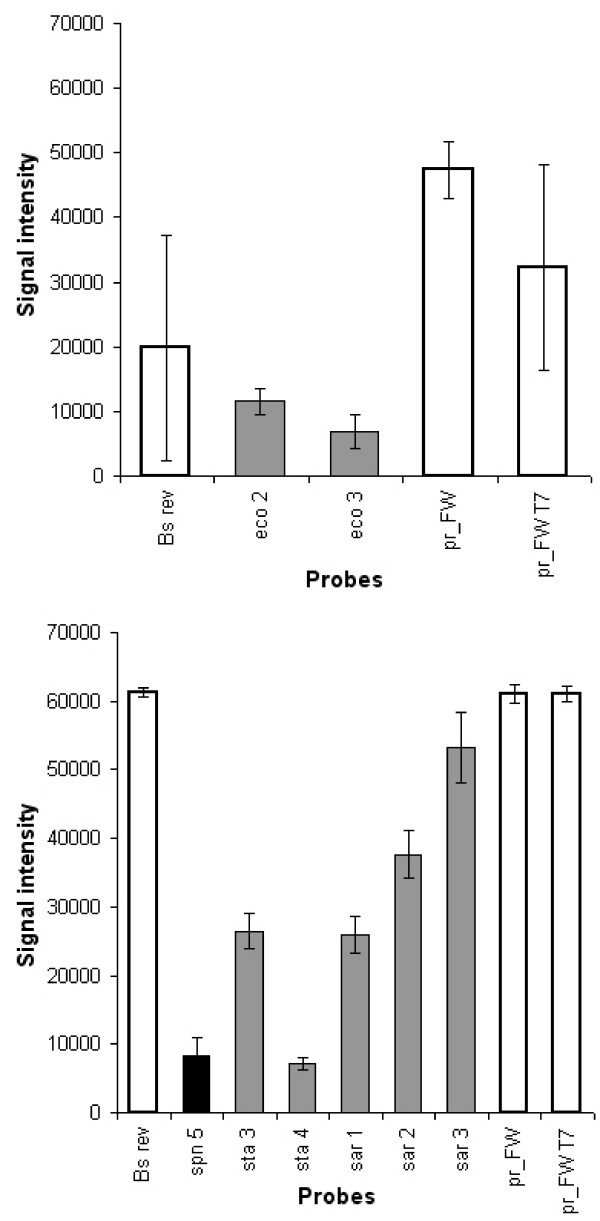
Examples of individual hybridisation results. Experiments using *E. coli *(A) and *S. aureus *(B) were done as dilution series near their limit of detection. *E. coli *shows a much lower detection limit of 10 bacteria per assay than *S. aureus *with 10^3 ^bacteria per assay. Even at lowest sensitivity level specific hybridisation patterns can still be obtained. Grey, black and white bars represent specific and non-specific signals as well as positive controls (BSrev is the hybridisation control and pr_FW and pr_FW T7 are PCR amplification controls). Error bars represent the mean of 6 replicate spots on the microarray. These figures only display intensities of spot signals which were flagged positive automatically by the GenePix Software. However for statistical analysis (quantile normalisation) all signals were evaluated. Results were controlled by visual inspection of image files.

Identification based on microarrays was 100 times more sensitive than the agarose gel evaluation demonstrated. Specific and reproducible signals down to 10 bacteria per assay could be achieved for *E. coli*. Analysis of *Staphylococcal *cultures revealed the highest detection limit within the group of gram positive bacteria with about 10^3 ^cells necessary per assay to see signals on the microarray (see Fig. 2). Sensitivity studies were carried out with other gram negative and positive species and found to be similar to those mentioned before. *C. freundii *and *K. pneumoniae *had a detection limit of 10^2 ^bacteria per assay whereas *E. faecalis *and *S. epidermidis *were detectable only at 10^3 ^cells per assay. This difference in sensitivity can be ascribed to less efficient cell lysis due to the presence of a persistent cell wall and the presence of a thermostable DNAse in the Staphylococcal proteome [34]. The adaptation of the protocol to different cell lysis steps or an additional enzymatic treatment can certainly further improve the detection limit.

### Parallel detection of pathogens

**Figure 3:**
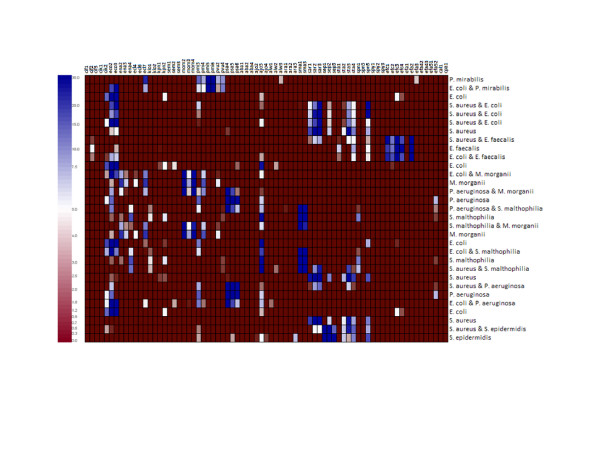
Comparison of different parallel identifications of pathogens. Heatmap was drawn after hierarchical clustering. Each target combination was compared with hybridisation results of single cultures under equal experimental conditions. Rows correspond to probes and columns correspond to hybridisations. Colours correspond to signal values. Blue displays high signal value and red no signal value.

**Figure 4:**
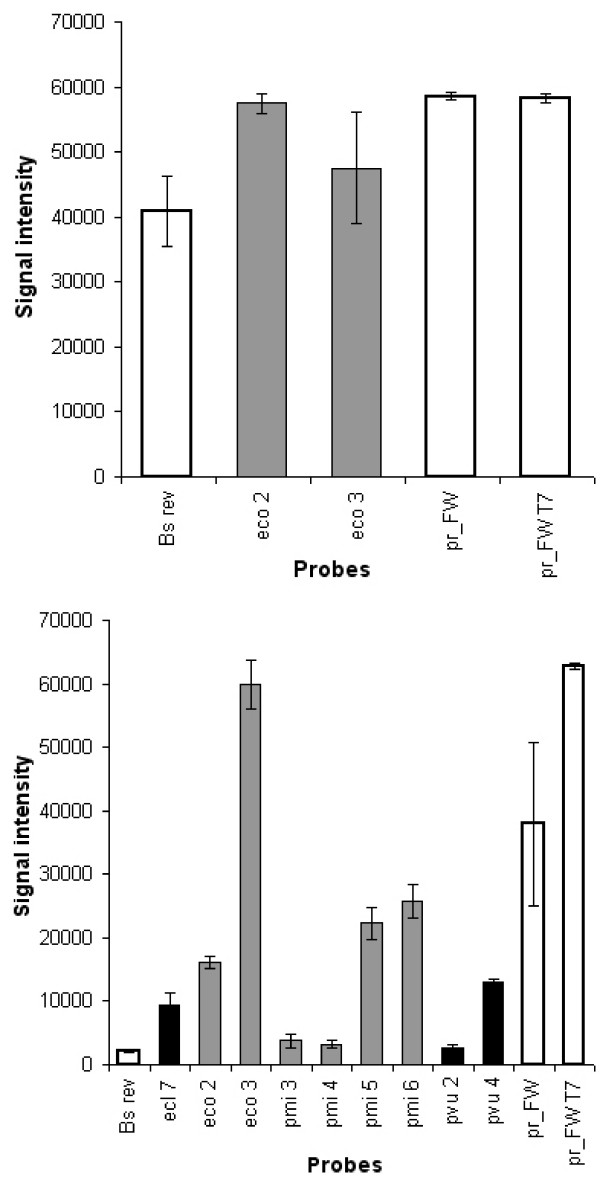
**A**: Hybridisation signals of *E. coli *isolated from whole blood. Despite the great background of human DNA in blood no interference (non-specific signals would be displayed in black) were observed. These results prove the high specificity of the protocol for clinical pathogens. Specific signals are shown as grey and positive controls as white bars. **B**: Isolation of bacterial DNA from blood spiked with *E. coli and P. mirabilis*, simulating a multi-microbial infection. It clearly shows the possibility of parallel detection of different microbes even from one human blood sample. Abbreviations of probe names are listed in table 1. Grey, black and white bars represent specific and non-specific signals as well as positive controls. These figures only display intensities of spot signals which were flagged positive automatically by the GenePix Software. However for statistical analysis all (both negative and positive flagged) signals were evaluated. Results were controlled by visual inspection of image files.

The densities of bacterial suspensions were adjusted as described in Materials and Methods and equal amounts were added to single species and double species experiments. The hybridisation results of combinations of different strains were compared to those of single strains. The multiple microbial assays produced a signal pattern that matched the compounded signals of single species hybridisations (see Fig. 3). Due to these results a clear differentiation of species in a multiple microbial infection is possible. Some experiments were carried out based on spiked blood confirming the results of pure cultures (Fig. 4B). Parallel detection showed that most probes generated weaker signal intensities when two pathogens were present in one sample. This fact would lead to a lower sensitivity when polymicrobial samples are handled which enhances the risk of false result interpretation near the detection limit.

### Hybridisation of blood sample isolates

Starting from bacterial DNA isolated from blood PCR and labelling protocols were optimised with respect to reducing interference of blood components. Addition of glycerol and betaine reduced non-specific amplification during the PCR and labelling steps in spite of large amounts of residual human DNA. By this means the yield of specific PCR product was also clearly increased resulting in equal specificities as with cultured microbes. No cross-hybridisation provoked by human DNA was observed (Fig 4A). Similar results were obtained when detecting combinations of single microbes simulating multiple microbial infections as already described above. The obtained signal patterns were as specific for the added strains as those from single species microarray hybridisations (Fig. 4B).

The sensitivity of the method was determined by providing a ten-fold dilution series in 10 mL spiked blood. Detection limit was found to be as low as 10 bacteria per mL whole blood. However, as observed with pure cultures the sensitivity of gram positive bacteria is much lower, e.g. 10^5 ^per mL blood for *S. aureus*.

### Normalisation

Normalisation is an important aspect of all microarray experiments. Usually it requires a set of probes which are expected to give a constant signal throughout all hybridisations. In the present application there are no endogenous genes or sequences to suit this requirement. Therefore the only other option would be to spike known amounts of sequences similar to the 16S rRNA gene into bacterial suspension before cell lysis or directly into the DNA extract. However, spike controls are known to be potentially unreliable due to inaccuracies in nucleic acid quantitation and pipetting errors. Also, there could be competitive effects between the spike oligonucleotide and the target 16S gene during PCR, thus introducing a bias. Therefore a quantile normalisation approach was chosen, based on the assumption that each array should have a number of probes which give a positive signal (corresponding to the pathogen present in the sample) and the rest of the probes a low (or no) signal. This algorithm is a between-array normalisation approach which shifts the signal density of each hybridisation towards an average density across all hybridisations [35].

### Classification

**Figure 5:**
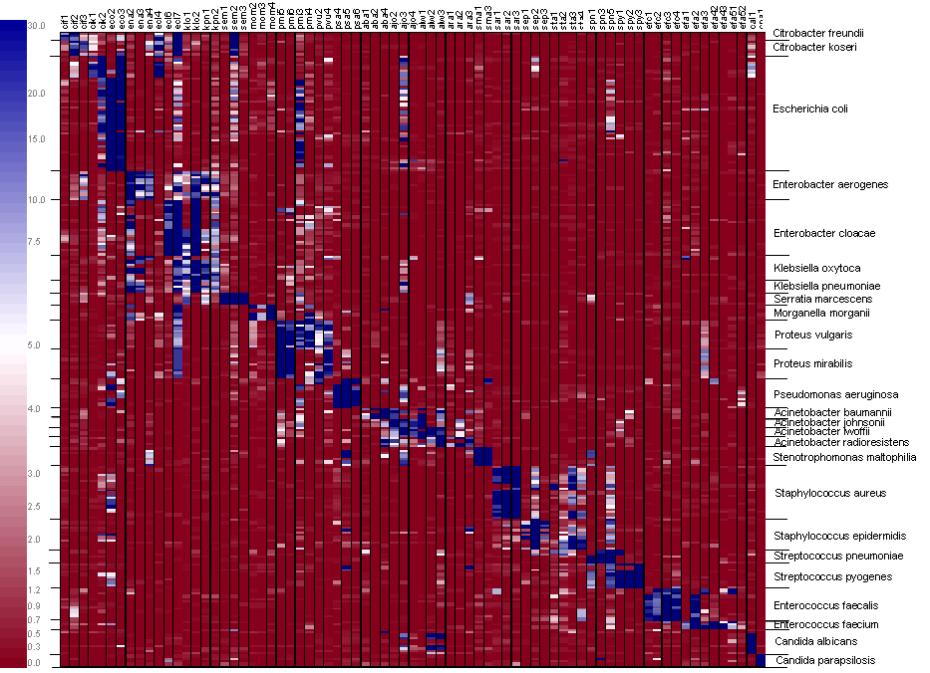
Results of all hybridisation experiments displayed as a heatmap. Columns correspond to probes and rows correspond to hybridisations. Colours correspond to signal values so that red indicates no signal succeeding to white for low signal strengths to blue indicating strong signal values (shown by the colour bar on the left side of the figure). The coefficient of variation of the different assays was already given along with the table of normalised signal values.

Fig 5 shows the clear clusters of hybridisations as well as of probes. Although each probe was designed to bind to one specific pathogen, the heatmap shows that some probes are very specific to one species while others yield signals for a wider range of different organisms and a few probes do not show any specific signal at all. A classical approach would be to evaluate each probe set across all hybridisations and define a signal threshold e.g. by ROC analysis [36] to distinguish positive from negative signals. However, since some probes show cross-hybridisation between species or even genera, this would not only lead to problems with specificity, but would also mean a loss of information contained in the cross-hybridisation patterns. A machine learning approach was used to classify a hybridisation pattern by similarity to hybridisations with known organisms. The k-Nearest Neighbour (k = 1) method was used and validated in a leave-one-out cross-validation approach. At genus level, all 241 hybridisations were classified correctly and 96.7% at species level. In 6 cases the species *P. mirabilis *and *P. vulgaris *and in 2 cases *A. baumannii *and *A. radioresistens *were confounded.

These misclassifications are not surprising, taking into account the high sequence similarity between the respective organisms. Possibly some of the misclassifications are due to low numbers of hybridisations in the test set, and could be avoided by adding more experiments to the test set.

## Discussion

The present microarray for the identification of blood isolates is so far the first microarray-based molecular diagnostic tool to identify a wide range of clinically relevant bacteria and yeast directly from blood in an appreciated period of time. Another similar approach applying isolates of positive blood cultures has been published previously [37]. The authors of this article introduce 19-mer 16S rDNA signature oligonucleotides which were spotted on Ta_2_O_5_- coated microarrays in order to allow evanescent-waveguide readout. Hybridisation data were analysed using hierarchical clustering analysis and pattern recognition appyling a decision tree.

The combination of PCR amplification with microarray hybridisation represents a powerful tool for pathogen identification. It exceeds conventional technologies in speed while performing at an extremely high specificity. Analysis of 16S rRNA genes has been reported before to allow a more robust, reproducible, and accurate testing than phenotypic methods [38]. Over 27000 sequences have been analyzed in the ARB software package to design species- and genus-specific microarray probes and calculate the hybridisation behaviour of these probes to target DNA from the respective bacterial species. Predicted and experimental values showed strong correlation. Short oligonucleotide probes ranging between 20 and 30 mer were used for this study because they allow distinct discrimination of single nucleotide differences under stringent conditions. Their hybridisation properties are fundamentally different compared to long oligonucleotide probes which usually have a length of 50 to 80 mer thus showing higher binding capacities and thereby leading to higher detection sensitivities on a microarray. However, the threshold of differentiation of long oligonucleotide probes is at best 90 % sequence similarity, which makes them not suitable for species identification based on the 16S rRNA marker gene.

Other marker genes were considered as potential targets for probe design especially some genes already known for their elevated sequence variability that might allow better discrimination of members of the family *Enterobacteriaceae*. However, an eminent advantage of the 16S marker gene is its good characterisation and the online availability of thousands of sequences. Furthermore in the meantime there is an ARB databank for free downloading on the homepage of the Technical University Munich which contains more than 50000 complete and aligned 16S rRNA sequences including phylogenetic relations calculated by the neighbour joining method. Additional sequences can be downloaded from any online database or manually added to the ARB database. This way the accession to 390 973 sequences of 16S rRNA genes is possible through the Ribosomal Database Project II  (release 9.52; July 5, 2007). With our approach we showed that this marker gene is well suited to discriminate even very closely related species. It was further demonstrated that analysis based on ribosomal RNA can be performed in presence of a strong background of human DNA.

Taking all the considerations into account there still remain two possible approaches for probe design. First the use of long oligonucleotide probes which allows high sensitivity. However, for equal specificity the application of several marker genes would be necessary to identify the whole panel of microbes. Such an approach necessitates the realisation of a multiplex PCR that might attenuate benefits in sensitivity of long probes. Further distinct species discrimination within the family *Enterobacteriaceae *would still be difficult. On the other hand the employment of short probes targeting solely the 16S rRNA gene also allows good discrimination and even species identification by pattern recognition within very closely related species. Applying stringent conditions this approach even allows detection of single nucleotide differences (compare figure 1 with Additional file 2). Furthermore the use of the most characterised marker gene with the highest number of available sequences allows reliable *in silico *predictions based on experimentally obtained data.

Standard clinical identification procedures require 2 days and up to 5 days for microorganisms that are difficult to cultivate. In contrast to cultivation- and bacterial enrichment dependent methods microarrays enable not only a fast and accurate but also highly parallel identification of different microorganisms in one assay. The present protocol can be carried out within 6 hours from drawing blood to the presentation of results by an analysis software, when gel electrophoresis for PCR product confirmation and pre-hybridisation are carried out in parallel with labelling reaction. Most identifications were realised analysing 6 different samples and 1 positive as well as 1 negative control. Therefore 8 samples had to be proceeded in parallel. The DNA preparation lasted 1.5 hours, the amplification reaction 2.5 hours, the labelling 50 min, the hybridisation 1 hour, the washing 10 minutes and the scanning also 10 minutes. The applied mastermixes and buffers were prepared prior to the analysis in large amounts so that each solution was ready to use. Thus diagnosis can be obtained within a working day when blood is collected from the patient in the morning. The current assay time of 6 hours can be easily reduced by 2 hours using meanwhile commercially available fast PCR devices (PCR run time: 20 mins). Online fluorescence scanner or electrode chip based systems could be applied for faster assay performance. A further reduction in assay time can be achieved when DNA isolation from blood is done as an automated and integrated process. Such systems are currently being tested in the clinical practice. The shorter duration would allow the realisation of several detection runs per day. Additionally, the handling steps would thus be reduced thereby reducing the danger of infection for personnel performing the assay.

Furthermore compared to cultivation-based identification procedures DNA based methods have the advantage to enable even the detection of static or dead cells before genome degradation e.g. in the case of administration of antibiotics when no further growth in culture can be observed [27]. However DNA-based detection methods are not able to distinguish between alive and dead cells which might lead to misguiding therapeutic conclusions since initial antibiotic treatments are always applied prior to first blood withdrawal. In the case of multiple infections, where one species is already eradicated by antibiotics, the other species could be underestimated due to a high amount of DNA from the non-viable bacteria.

Most studies on nucleic-acid based identification of blood-borne pathogens, including multiplex-PCR, real-time PCR and microarrays, were so far restricted to blood cultures and mostly to the identification of selected members of the *Enterobacteriaceae *family or gram positive bacteria like *Staphylococcus *and *Streptococcus *[3,17,39-44]. The application of microarrays for pathogen identification of clinical specimens has already been proposed earlier. Amongst published tools are arrays for upper respiratory tract infections, urinary tract infections, human faeces analysis and blood stream infections. Microbial detection has been achieved applying different marker genes because of known inconvenience of 16S rRNA due to high sequence similarities between closely related species. The suitability of 23S rRNA, the topoisomerases *gyrB *and *parE *has been proven for distinct species discrimination [4,30,31,42] The small and large subunit rRNA genes, virulence factors and antibiotic resistance determinants have been successfully applied as markers for the detection of bloodstream infections [43,45]. However, all microarray based studies were carried out so far with clinical specimens isolated from blood culture that partially neutralises the main advantage of identification by molecular methods. On the other hand direct detection from whole blood samples implicates higher detection limits. A general limitation of microbial detection microarrays compared to blood cultures is the generation of false negative results for species with no specific probe within the panel. This study was carried out with whole blood and it was shown that no cross-hybridisation is caused by human background DNA (Fig. 4). The developed probe panel further covers the species most frequently associated with bloodstream infections and can easily be extended. The applied normalisation has the advantage that no more control is needed for correct classification of the outcomes. Each result can be added to the classifier leading to even more precise calculations.

The present pathogen identification microarray is to our knowledge the first microarray assay for the parallel identification of a total of 23 blood-born bacterial species as well as 2 clinical relevant *Candida *species which enables pathogen identification down to 10^1 ^CFU/mL blood without any preculturing. Using this microarray for pure cultures detection limits in the range of 10^1 ^and 10^3 ^bacteria per assay were achieved, whereas the limit of detection for bacteria in spiked blood was found to be 10^1 ^to 10^5 ^bacteria per mL blood depending on the targeted microbes. Since even in blood of severe sepsis patients only 10^2 ^viable pathogenic cells per mL blood can be found this high detection limit is a clear shortcoming of this method implicating false negative results or the risk of detection of mostly dead cells thus leading to misguidance. The higher LOD of spiked blood samples compared to pure cultures results from PCR inhibitory components in blood [46,47]. Additional DNA purification can reduce the amount of these inhibitors, but high levels of residual human DNA still render lower LOD difficult. Due to high sequence similarities of the 16S rRNA gene short oligonucleotide probes were used in order to increase specificity. However, such an improvement implicates a reduction of sensitivity which also leads to strong signal variations near the limit of detection. In our study this problem could be overcome by microarray result evaluation by the developed pattern recognition which quantities and normalises signal strengths. But in routine application this fact would display a high risk of result misinterpretation near the detection limit.

Different approaches to increase signal intensity and to further reduce the LOD of microarray analysis may be applied to this test. One possibility is the usage of a rotating microchamber for supporting the stirring of the hybridisation mix [48-51]. Other improvement strategies concern the choice of probes for detecting pathogens. This includes the introduction of poly-T spacers to DNA probes to avoid steric hindrance during hybridisation, investigating different lengths of probes or enlarge the probe selection to several thousands in order to produce specific patterns and to cover a wide range of identifiable organisms [52-54].

The presented pathogen microarray includes 76 DNA probes with an average length of 20–30 bp, all derived from the prokaryotic and eukaryotic small-subunit ribosomal RNA gene. Due to high sequence similarities in the 16S rRNA gene especially among members of the *Enterobacteriaceae *group, it was evident that not all designed DNA probes would give 100% species-specific signals on the microarray. However, we could overcome this problem by taking advantage of the species-specific patterns of signals obtained after microarray hybridisation. Applying a supervised k-Nearest neighbour (k = 1) classification method all of the tested bacteria and yeasts were identified correctly at the genus level and 96.7 % at the species level. High 16S rDNA sequence similarity caused only misclassification in case of *P. mirabilis *and *vulgaris *and *A. radioresistens *and *baumanii*, respectively.

Based on this microarray 25 different microbes of bloodstream infections are identifiable. However this panel may be expanded by further organisms as new pathogens appear in clinical routine and slight variants are observed when organisms are isolated from clinical specimens in different regions [4]. Further microorganisms could be *Staphylococcus lugdunensis*, streptococci like *S. agalactiae*, *Salmonella spp*., *Aeromonas spp*. or *Candida krusei *which could be easily included in the current microarray by designing species- and genus specific probes for them.

A database was established serving as a classifier for the applied statistical method. This analysis method includes pattern recognition and machine learning algorithms. The chosen algorithm K-nearest-neighbour method executes an accurate identification within a fully automated platform. Moreover a software package is under development which includes the flexibility of subsequent addition of single probes, individual species, groups of species or even an exchange of the whole classifier. A possible enlargement of the classifier by addition of further hybridisation results increases the specificity of identification, by reducing misinterpretation possibility due to false negative signals or cross hybridisations (especially for *Proteus *and *Acinetobacter *species). Such a software allows automatic processing of microarray image files and will retrieve genus and species level identification. Additionally, recommendations of appropriate antibiotic treatments can be given from the statistical assessment of periodically updated information on antibiotic resistances. However, such guidance only relies on past data of statistical records. The most appropriate assistance would be gained based on antibiotic resistance determination by detection of the genetic determinants. Such information can only be obtained by the realisation of multiplex PCR which would further reduce the sensitivity of the method.

Since seven percent of all bloodstream infections are polymicrobial [9], the probe pattern and the classification algorithm were also tested for randomly selected dual bacterial combinations. Signal patterns from multiple microorganisms detection could be predicted from single microbe signals. In each case both pathogens present in the sample could be correctly identified. Negative controls of unspiked blood gave negative PCR amplification and hybridisation results. This confirms the absence of bacteria or bacterial DNA in the blood of healthy humans [55,56].

The combination of microbial identification with clinical antibiotic resistance determination reveals a considerable potential. DNA microarrays have already been adopted for detection of quinolone-resistant *E. coli *or for the genotyping of TEM beta-lactamases [57,58]. An antibiotic resistance microarray specifically targeted to blood-born pathogens is under development in our group following the successful establishment of a first prototype array.

## Conclusion

In this study we have developed a rapid and sensitive method for DNA based identification of clinically relevant pathogens that cause bloodstream infections. The microarray method has been shown to detect and identify pathogens down to concentrations of 10 bacteria per mL within 6 hours. Relying on the analysis of signal patterns the assay specificity was determined to be 100 % at genus level and more than 96.7 % at species level. However, depending on clinical specimen the isolation protocol must be adapted in order to optimise microbial DNA yield and to assure maximum safety to operating personnel. But the work also shows that a microarray based on the 16S rRNA marker gene is a potentially powerful identification tool for routine clinical laboratory diagnostics. Additionally the range of identifiable organisms can easily be extended to new pathogens and also multimicrobial infections can be considered.

## Methods

### Samples – Reference Strains

All reference strains tested in this study were obtained from the American type culture collection (ATCC) or the "Deutsche Sammlung für Mikroorganismen und Zellkultur" (DSMZ). In addition to the reference strains probe specificity and sensitivity were also tested with clinical isolates which had been identified by classical microbiology methods. For long term storage all bacterial strains were kept as 50 % glycerol stocks at -80°C. For most of the experiments pure cultures of a certain number of bacteria per mL were used which were obtained by cultivating the respective microbe in Caso bouillon overnight at 37°C and finally adjusting the microbe concentration per mL using a Mc Farland standard # 0.5. All experiments and the validation of the microarray were carried out using clinical isolates and only some culture collection strains. Clinical isolates were directly identified in the hospital laboratories based on commercial microbial identification techniques subsequent to blood culture. Cultures were delivered in cryovials, Microbank from Pro Lab Diagnostics (Neston, United Kingdom). Microarray testing was performed on *Escherichia coli *(ATCC 35218, clinical isolates: 11063, 15130, 81617, 68933, 68307), *Enterobacter aerogenes *(DSMZ 30053, clinical isolate: 12676), *Enterobacter cloacae *(clinical isolates: 26385, 79232, 93840, 12720, 74892), *Klebsiella pneumoniae *(clinical isolates: 25809, 85813, 26385, 13253), *Klebsiella oxytoca *(clinical isolates: 26785, 26384, 73739, 26786, 96633), *Citrobacter koseri *(DSMZ 4595), *Citrobacter freundii *(clinical isolates: 80324, 73489), *Staphylococcus aureus *(ATCC 6538, ATCC 25923, ATCC 29213, clinical isolates: 83799, 82913, 73237, 12998), *Staphylococcus epidermidis *(ATCC 14990, clinical isolates: 73711, 35989, 80320, 13000, 77504, 79510), *Enterococcus faecalis *(ATCC 29212, clinical isolates: 49395, 81239, 83776, 27520), *Enterococcus faecium *(DSMZ 20477), *Streptococcus pneumoniae *(DSMZ 25500), *Streptococcus pyogenes *(ATCC 19615, clinical isolate: 10388), *Proteus mirabilis *(ATCC 14153, clinical isolates: 26786, 27761, 97656, 71913), *Proteus vulgaris *(DSMZ 13387, clinical isolate: 80196), *Serratia marcescens *(DSMZ 30121), *Morganella morganii *(DSMZ 6675, clinical isolate: 12615), Pseudomonas aeruginosa (clinical isolates: 26178, 12950, 26535, 68961, 74352), *Stenotrophomonas maltophilia *(DSMZ 50170, clinical isolates: 26394, 26396), *Acinetobacter baumannii *(DSMZ 30007), *Acinetobacter lwoffii *(DSMZ 2403, clinical isolate: 75496), *Acinetobacter radioresistens *(DSMZ 6976), *Acinetobacter johnsonii *(DSMZ 6963), *Candida albicans *(ATCC 10231, clinical isolate: 21179, 27184, 96917, 96635), *Candida parapsilosis *(clinical isolate: 4344). Note: Strain numbers mentioned above without any specification of microorganism collection are clinical isolates.

### Oligonucleotide probe design

Probe design and analysis were performed with the ARB software package [59]. Selected ribosomal DNA (rDNA) sequences of pathogenic bacteria and yeasts were downloaded from the GenBank of the NCBI homepage and uploaded to the ARB software package to create a database comprising over 27.000 16S rDNA sequences but also over 7000 18S rDNA sequences to detect possible mismatches with eukaryotic sequences. After the new sequences had been aligned to the preexisting database [release June 2002] a phylogenetic tree was calculated using the neighbour joining method. Probes were designed for species and selected genera based on the results of the ARB software using the Probe Design function including alterable parameter settings such as probe length (20 bases), maximum non group hits, G+C content, melting temperature and minimum hairpin loops.

Probe sequences were tested for duplex and hairpin formation and melting temperature with the software "Oligo". Every single probe was optimised regarding melting temperature and duplex formation by deleting or adding bases. Final probe sequences were checked with the Probe Match function in ARB. Each generated hybridisation table with sequences of organisms matching to any single probe served as input for CalcOligo [60], a software for weighted mismatch calculation. Mismatches were weighted according to experimentally determined formulas. (see table 2 and table 3).

Single mismatches of each probe were added to yield a total weighted value for each species. Values were arranged to generate an *in silico *calculated hybridisation matrix, sequentially tabulated in an Excel spreadsheet.

### Microarray preparation

Synthesised oligonucleotide probes were obtained from VBC Genomics (Vienna, Austria). At the 5' end of each oligo 5 thymine residues were added as spacer molecules. In order to ensure covalent linkage to the reactive aldehyde group on the microarray surface (CSS-100 Silylated Slides, Cel Associates, Texas) probes were 5' amino-modified. Probes were printed at different concentrations (50 μM, 20 μM and 10 μM in 3× SSC and 1.5 M betaine monohydrate) onto the silylated glass slides by the contact arrayer Omnigrid from GeneMachines (San Carlos, California) while the adjusted air humidity was between 55 and 60 %. 6 replicates of each probe were printed per microarray. Spotting was carried out with SMP 3 pins (TeleChem, Sunnyvale, California) leading to a spot size of 100 μm diameter.

A hybridisation control probe (5' -TTA AAA CGA CGG CCA GTG AGC) was spotted on the array applying the same conditions as used for the target capture probes.

### DNA isolation

Blood samples were taken by sterile withdrawal into a 10 mL K3E tube (BD Vacutainer Systems) from Becton Dickinson (Oxford, UK). Bacteria were spiked into blood by adjusting the appropriate density using McFarland standard # 0.5 and transfering the correct volume or dilution into 10 mL whole blood. For preliminary blood cell lysis 3 mL of Tris-EDTA (TE) (10 mM Tris, 1 mM EDTA, pH 8) were added, mixed and centrifuged at 10000 g for 10 min. The supernatant was discarded and a pellet volume of about 0.75 to 1 mL remained in the Falcon tube. TE lysis was repeated by addition of 10 mL TE buffer, vigorously vortexing and centrifugation at 10000 g for 10 min. The supernatant was than discarded to obtain a cell pellet of a volume of about 50 μL which was resuspended in physiological NaCl and carefully transferred to the top of a Percoll (Amersham Biosciences, GE Healthcare, Uppsala, Sweden) solution. Physical density of Percoll was adjusted to 1.05 g/cm^3 ^according to the manufacturer's instructions. The density centrifugation was carried out at 1500 g for 20 min. The supernatant was discarded and the pellet was rinsed twice with physiological NaCl in order to remove residual Percoll. The remaining pellet was resuspended in 50 μL of distilled water and cell lysis was done by heating the suspension to 95°C for 15 min. The lysed suspension was centrifuged at 10000 g for 10 min to remove cell debris and to obtain the released DNA. The supernatant, containing the resulting DNA, was transferred to a new tube.

### DNA amplification

For DNA amplification a multiplex PCR was performed targeting the small ribosomal subunits of eukaryotes and prokaryotes. The 16S rRNA gene was PCR amplified employing the forward primer 27 T7 (5'- TAA TAC GAC TCA CTA TAG AGA GTT TGA TCM TGG CTC AG) and the reverse primer 1492 (5'- TAC GGY TAC CTT GTT ACG ACT T) (VBC Genomics, Vienna, Austria) (0.3 nM in PCR mixture) [61]. The forward primers contained the T7 promoter site (5'-TAA TAC GAC TCA CTA TAG -3') at their 5' end, which enabled T7 RNA polymerase mediated *in vitro *transcription using the PCR products as templates for direct comparison of different labelling methods [62]. Candida species were identified by prior amplification of the 18S rRNA gene with the primers CanFW (5'- TCC GCA GGT TCA CCT AC) and CanRev (5'- CAA GTC TGG TGC CAG CA) [63].

Bacteria in 10 mL whole blood served as target scenario for optimisation of generation of full length 16S rRNA amplicons. Efficiency of the PCR was optimised with bacterial DNA isolated from 1 mL blood by varying the concentrations of different components and adding PCR enhancers. Most efficient amplification was found by addition of 5 μL DNA extract. Optimal conditions for a 25 μL PCR reaction mixture were: 3 U Taq DNA polymerase (Invitrogen, Carlsbad, California), 2.5 μL 10× PCR-buffer, 0.5 mM each dNTP, 2 mM MgCl_2_; 10 % glycerol and 0.5 % betaine.

PCR cycling included an initial denaturation step at 95°C for 5 minutes, followed by 40 cycles of 95°C for 30 sec, 55°C for 1 min, and 72°C for 1 min using a Biometra T3000 Thermocycler (Goettingen, Germany). Temperature cycles were terminated at 72°C for 10 min to complete partial amplicons, followed by storage at 4°C until further usage.

Successful amplification was confirmed by resolving the PCR products on a 1.5 % agarose gel (SeaKem, Biozym, Vienna, Austria) with ethidium bromide in TBE buffer (0.1 M Tris, 90 mM boric acid, 1 mM EDTA) (Invitrogen, Paisley, UK).

### Labeling

The primer extension method showed the best sensitivity and specificity and was therefore used as standard labelling method. 6 μL of PCR product were used for labelling in the primer extension reaction mix, which contained each 0.9 mM forward primer 27 (16S rRNA) and CanRev primer (18S rRNA), 1.5 U Vent (exo) polymerase (New England Biolabs, Ipswich, UK), 3 mM MgSO_4 _and 50 μM of dATP, dGTP, dTTP, 25 μM of dCTP and 25 μM Cy5-dCTP. The reaction mix was cycled 25× at 95°C 60°C and 72°C each 20 sec followed by a final extension step for 5 min at 72°C. Temperature cycles were preceded by 3 min incubation at 95°C.

### Hybridisation

Prior to hybridisation the microarray slides were pre-treated with blocking buffer (cyanoborohydride buffer: 20 mM Na_2_H PO_4_, 10 mM NaH_2_PO_4_, 200 mM NaCl, 50 mM NaBH_3_CN) at room temperature for 30 minutes in order to inactivate reactive groups on the slide surface.

The hybridisation mixture had a final volume of 30.3 μL. It contained 24 μL of the labelled DNA reaction mixture and was adjusted using 20× SSC and 10% SDS to a final concentration of 4× SSC and 0.1% SDS. The suspension was denaturated at 95°C for 5 minutes and finally a hybridisation control (BSrev: 5' end Cy3-labeled oligonucleotide sequence: AAG CTC ACT GGC CGT CGT TTT AAA) was added to a final concentration of 0.15 nM. A total volume of 22 μL was transferred to a cover slip (22 × 22 mm) and subsequently applied to the microarray surface. Hybridisation was realised at 65°C in a vapour saturated chamber for 1 h. Slides were washed in 2× SSC and 0.1 % SDS for 5 minutes followed by 0.2 × SSC for 2 minutes and 0.1× SSC for 1 minute. Slides were dried by centrifugation at 900 g for 2 minutes.

### Signal detection and data analysis

Slides were scanned at a resolution of 10 μm with an Axon Genepix 4000A microarray scanner (Axon, Union City, California) at equal laser power and sensitivity level of the photomultiplier (650 pmt) for each slide. Therefore absolute and relative signal intensities presented for independent experiments are directly comparable. Obtained images were analyzed using the Genepix software and the resulting gpr-files were used for further analysis.

### Statistical evaluation

Data analysis was done in R [64] using the packages *limma, affy, stats *and *class*. Datasets consisted of 241 hybridisations done on 3 different layouts of the pathogen identification microarray. The different layouts shared 76 probes; these were used in the analysis. All other probes were disregarded. Each pathogen was represented by 2–5 different probes with different sequences. To increase robustness, probes were spotted 6 times on the array.

Each hybridisation was represented by one gpr file, all of which were collectively stored as RGList objects in R. Signals were normalised using quantile normalisation from the *affy *package. Medians of the 6 spot-replicates were used for supervised k-Nearest neighbour (k = 1) classification method. The classifier was validated in a leave-one-out cross-validation approach.

## Authors' contributions

CN was responsible for organisation and concept development. LB contributed to probe design and method construction. EP and AMH supplied practical clinical support. AK supported statistical evaluation of microarray data together with KV who developed the microarray pattern recognition approach. RP and HWM were responsible for design of experiments, evaluation of results and method development. All authors read and approved the final manuscript.

## Supplementary Material

Additional file 1 *In silico *hybridisation matrix. Matrix predicting hybridisation behaviour of the designed microarray probes (horizontally plotted). The *in silico *hybridisation matrix was generated with the Probe Match function in the ARB software package and the CalcOligo software. Click here for file

Additional file 2 Hybridisation results of all experiments. Single signal results of all hybridisations are summarised in a datasheet. The supplied outcomes are the signal medians obtained after quantile normalisation. Click here for file
